# Associations between multiallelic genetic variants and ADRD‐related proteinopathy

**DOI:** 10.1002/alz70855_105861

**Published:** 2025-12-24

**Authors:** Yuriko Katsumata, Khine Zin Aung, Xian Wu, Inori Tsuchiya, Lincoln MP Shade, Erin L. Abner, Peter T Nelson, David W. Fardo

**Affiliations:** ^1^ Department of Biostatistics, College of Public Health, University of Kentucky, Lexington, KY, USA; ^2^ College of Public Health, University of Kentucky, Lexington, KY, USA; ^3^ University of Kentucky College of Medicine Department of Epidemiology, Lexington, KY, USA; ^4^ University of Kentucky College of Medicine, Sanders‐Brown Center on Aging, Lexington, KY, USA

## Abstract

**Background:**

We recently reported genetic associations with dementia‐related proteinopathies. Using multidimensional generalized partial credit modeling, we constructed three continuous latent variables, corresponding to TDP‐43, Ab/Tau, and a‐synuclein related neuropathology endophenotype scores. However, we have not evaluated multiallelic sites, which are genetic variants with mutations that are more complex than simple nucleotide variation where conventional genetic coding (e.g., 0, 1, and 2 alleles) cannot be used. Here, we perform genome‐wide multiallelic variant analyses using whole genome sequencing (WGS) data generated by Alzheimer's Disease Sequencing Project (ADSP).

**Method:**

Participant data were drawn from the National Alzheimer's Coordinating Center (NACC) neuropathology (NP) v10‐11 data (from the September 2023 data freeze) linked to ADSP WGS data. After filtering out low quality calling variants, we evaluated 672,004 multiallelic variants in total (chromosomes 1 to 22). We applied score tests under the generalized linear model framework (assuming the normal distribution) adjusted for sex, age at death, and the top three principal components and computed global *p*‐values.

**Result:**

The total of 392 participants who were included in the multidimensional generalized partial credit modeling had both NACC NP and ADSP WGS data. The top hitters that had suggestively significant (defined as *p*‐value of less than 1×10^‐5^) associations with the TDP‐43 pathology (i.e., limbic‐predominant age‐related TDP‐43 encephalopathy neuropathologic changes, LATE‐NC) related latent scores were variants located in chr18:35995261 on *RPRD1A* (global score test *p*‐value = 1.4×10^‐6^), chr7:132612663 on *PLXNA4* (global score test *p*‐value = 6.0×10^‐6^), chr6:54528730 in an intergenic region (global score test *p*‐value = 8.2×10^‐6^), and chr14:72531431 on *RGS6* (global score test *p*‐value = 9.5×10^‐6^).

**Conclusion:**

Some multiallelic variants were suggestively associated with TDP‐43 proteinopathy. *PLXNA4* has been reported as an AD‐associated gene in previous studies. Further analyses are required to confirm these associations using independent cohort data.

